# A Robust, Fully Automatic Detection Method and Calculation Technique of Midline Shift in Intracranial Hemorrhage and Its Clinical Application

**DOI:** 10.3390/diagnostics12030693

**Published:** 2022-03-11

**Authors:** Jiun-Lin Yan, Yao-Lian Chen, Moa-Yu Chen, Bo-An Chen, Jiung-Xian Chang, Ching-Chung Kao, Meng-Chi Hsieh, Yi-Ting Peng, Kuan-Chieh Huang, Pin-Yuan Chen

**Affiliations:** 1 Department of Neurosurgery, Keelung Chang Gung Memorial Hospital, Keelung 204, Taiwan; mailtomaxi@gmail.com; 2 School of Traditional Chinese Medicine, College of Medicine, Chang Gung University, Taoyuan 333, Taiwan; 3 Department of Radiology, Keelung Chang Gung Memorial Hospital, Keelung 204, Taiwan; chenyl0702@cgmh.org.tw; 4 Department of Neurosurgery, Linko Chang Gung Memorial Hospital, Taoyuan 333, Taiwan; u9601056@gmail.com; 5 AI Lab, Quanta Computer Inc., Taoyuan 333, Taiwan; okmegy@hotmail.com (J.-X.C.); eimafant@gmail.com (C.-C.K.); stanley.hsieh@quantatw.com (M.-C.H.); tina.peng@quantatw.com (Y.-T.P.); mark.huang@quantatw.com (K.-C.H.)

**Keywords:** intracranial hemorrhage, midline shift, automatic detection, CT

## Abstract

A midline shift (MLS) is an important clinical indicator for intracranial hemorrhage. In this study, we proposed a robust, fully automatic neural network-based model for the detection of MLS and compared it with MLSs drawn by clinicians; we also evaluated the clinical applications of the fully automatic model. We recruited 300 consecutive non-contrast CT scans consisting of 7269 slices in this study. Six different types of hemorrhage were included. The automatic detection of MLS was based on modified Keypoint R-CNN with keypoint detection followed by training on the ResNet-FPN-50 backbone. The results were further compared with manually drawn outcomes and manually defined keypoint calculations. Clinical parameters, including Glasgow coma scale (GCS), Glasgow outcome scale (GOS), and 30-day mortality, were also analyzed. The mean absolute error for the automatic detection of an MLS was 0.936 mm compared with the ground truth. The interclass correlation was 0.9899 between the automatic method and MLS drawn by different clinicians. There was high sensitivity and specificity in the detection of MLS at 2 mm (91.7%, 80%) and 5 mm (87.5%, 96.7%) and MLSs greater than 10 mm (85.7%, 97.7%). MLS showed a significant association with initial poor GCS and GCS on day 7 and was inversely correlated with poor 30-day GOS (*p* < 0.001). In conclusion, automatic detection and calculation of MLS can provide an accurate, robust method for MLS measurement that is clinically comparable to the manually drawn method.

## 1. Introduction

Intracranial hemorrhage caused by various conditions, including traumatic, spontaneous, or secondary disease, can lead to serious medical sequelae due to an increase in intracranial pressure (ICP), causing neural damage. However, the current gold standard for accurate assessment of ICP is the placement of invasive, intracranial ICP monitors. Several different techniques have been developed to evaluate intracranial pressure noninvasively [1]. One of the most widely used methods is the calculation of midline shift (MLS) from non-contrast computed tomography (CT) scans, especially in circumstances of increased ICP caused by localized mass lesions or parenchymal edema. The accessibility of MLS from CT and its clinical applications had been widely discussed [2]. The National Traumatic Coma Data Bank showed that seven out of eighteen patients who could talk and then deteriorated had a MLS greater than 15 mm [3]; other studies showed a continuous association of MLS and clinical outcome [4], and in some circumstances, an MLS greater than 5 mm could be an indication for surgical intervention [5].

The manual measurement of the MLS was proposed by the Brain Trauma Foundation [6]. First, the intracranial width was measured at the level of foramen Monro (A), and then the distance from the inner skull to the septum pellucidum was measured (B). The MLS can be calculated by (A/2) − B. Another approach is to measure the distance from a line joining the most anterior and posterior visible points on the falx to the farthest point on the septum pellucidum [7]. However, manually drawing the MLS can be time consuming and less reliable due to potential interrater variability. Therefore, several computer-aided or automated methods for MLS measurement have been developed to provide an objective robust method [2]. Liao et al. proposed a symmetry-based method to recognize the distance of the MLS by segmenting the brain into an upper segment, a lower immobilized falx, and a central curved segment [8]. Chen et al. combined symmetry and shape matching for the identification of MLS to correlate with the ICP level [9]. Other approaches, such as landmark-based approaches, were also proposed. Liu et al. developed an automatic detection method for anatomical landmarks for MLS measurement, especially for cases of large deformities [10].

In this article, we propose a fully automatic detection method for MLS based on the Keypoint R-CNN with the ResNet-FPN-50 backbone. We first compare this automatic detection method with MLS measured manually and MLS calculated by keypoints. Second, the MLS measured with different methods is correlated with the clinical outcome, including 30-day overall mortality, Glasgow Outcome Scale (GOS), Glasgow Coma Score (GCS), and ICP.

## 2. Materials and Methods

### 2.1. Patients Population

We retrospectively included 300 consecutively collected non-contrast brain CT scans from July 2019 to January 2020 from the same single CT machine (GE LightSpeed, slice thickness = 0.625 mm) in the emergent department in our institution. Indications for brain CT scans were for the diagnosis of intracranial hemorrhage, clinical deterioration of known intracranial hemorrhage (ICH) patients, and regular follow-up when clinically needed. All types of intracranial hemorrhage, including spontaneous ICH, traumatic ICH, acute subdural hematoma (ASDH), chronic subdural hematoma (CSDH), epidural hematoma (EDH), intraventricular hemorrhage (IVH), subarachnoid hemorrhage (SAH), or mixed type were included. All diagnoses were based on neuroradiologist reports. The exclusion criteria were low imaging quality, such as severe motion defects and failure of complete examination. Patients with intracranial foreign bodies, skull defects, or postoperative conditions were not excluded in order to test our algorithm in the real-world setting.

Measurement of the midline shift was done by three different methods. Manual MLS measurement of the maximal distance between the septum pellucidum from the midline was done by an attending neurosurgeon with 13 years of experience, a 5th year senior neurosurgical resident, and a research associate with 3 years of experience in clinical research. The second method is the manual calculation of the distance between keypoint detected by the algorithm (detailed as below section) and the fully automated MLS measurement.

Clinical medical records and radiologic reports were collected from all patients. Intracranial pressure was measured in those in whom an ICP monitor was inserted (intraparenchymal or ventricular monitor). Intraoperative and postoperative day 3 ICP data were recorded. The Glasgow Outcome Scale at 30 days was assessed according to the medical chart. This project was approved by the Chang Gung Medical Foundation

### 2.2. Neural Network Based Automatic Measurement of the Midline Shift

**Figure 1:**
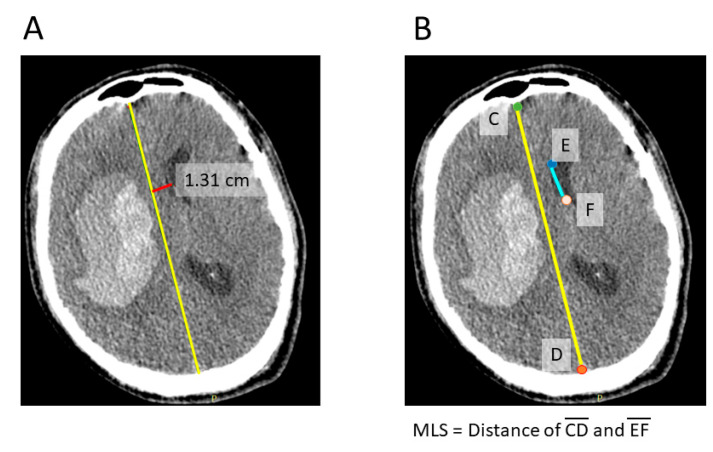
Figure 1 shows the method for manually drawing the midline shift (**A**). Firstly. identify the midline of the brain (yellow line), then measure the distance perpendicular to the septum pellucidum (red line). The manually selected keypoints were defined as shown in (**B**), including the anterior-most point of the anterior falx (**C**), the posterior-most point of the posterior falx (**D**), and the endpoints of the septum pellucidum (**E**,**F**). The MLS was calculated by measuring the distance between the middle point of line AD and line BC.

**Figure 2:**
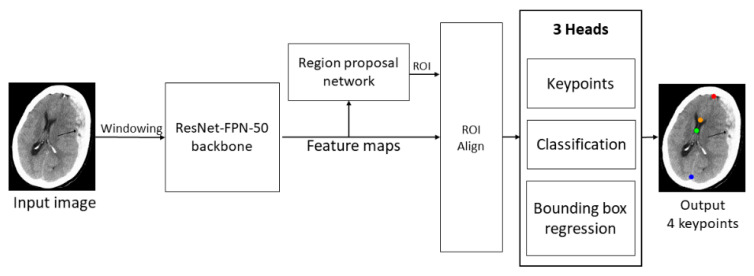
The architecture of Keypoint R-CNN. The Kepoint R-CNN contains ResNet-FPN-50 as a backbone, a region proposal network as a ROI generator, and three heads to realize the automatic detection of MLS.

The distance of the midline shift was assessed by using different methods (Figure 1), including manual drawing (Figure 1A), manual keypoint calculation (Figure 1B), and automatic MLS calculation (Figure 2). Manual drawing of the MLS was based on the distance between the falx and septum pellucidum in the most affected CT slice (Figure 1A). All CT scans were manually drawn by three authors (J.-L. Yan, K. Li, B.-A. Chen.) and were blinded to each other’s results. The keypoint for the calculation of the MLS was defined as the anterior end of the falx, the posterior end of the falx, the anterior end of the septum pellucidum near anterior commissure, and the posterior end of the septum pellucidum (Figure 1B). The keypoint MLS was defined as the distance between the middle point of the connection of the anterior and posterior falx and the midpoint of the septum pellucidum.

Automatic detection of the keypoints is achieved by Keypoint R-CNN derived from Faster R-CNN [11] and is a revision of Mask R-CNN [12]. The architecture of the Keypoint R-CNN was shown in Figure 2. The first stage is the region proposal network (RPN). It generates class-agnostic bounding boxes as proposals for the possible presence of instances. The second stage crops regions from the feature map extracted by the backbone network according to the proposals from the RPN and feeds them to three task heads, namely classification, bounding box regression, and keypoint detection, after applying region of interest (ROI) pooling to the feature crops. In contrast to Mask R-CNN, Keypoint R-CNN replaces the segmentation head with a keypoints prediction head, which predicts keypoints of the instance detected by the classification and detection head. The model is used to detect the 4 keypoints of each axial slice, and the MLS of the slice is then calculated by the keypoints. The greatest MLS of all slices is the MLS for the scan.

In the preprocessing phase, the axial medical images were resized to 512 by 512 pixels. A 3-channel image was created by stacking 3 spatially adjacent slices. Three windows whose levels and widths were (80, 200), (40, 380), and (600, 2800) were then applied to each channel, and the windowed channels were normalized to (0, 1).

The dataset for the learning task contains 300 scans consisting of 7269 axial images for 6 types of hemorrhage. The dataset was further split into 223 training, 31 validation, and 46 testing scans consisting of 5248, 769 and 1226 axial images. For the sake of fairness, scans of a patient were not distributed to the different dataset partitions (training/validation/testing), and scans in each type of hemorrhage were split to an 8:1:1 ratio.

In addition to manually annotated keypoints, the ground truth for training the Keypoint R-CNN also needs the bounding boxes for locating brain areas since it is a two-stage method (first detects brains and then detects keypoints within the brains). The ground truth bounding box is generated by scaling the line ADB with the factor of 1.2 and the width of the ground truth was generated by 0.8 times height.

To make the model robust enough in various situations, the following data augmentations were applied in training: random flipping along sagittal axis, random affine, and random erasing, and metal artifact simulation [13].

The model was trained on a NVIDIA V100 GPU for 200 epochs, with an initial learning rate of 0.0001 which was divided by a factor of 10 after 100 epochs. The batch size is 16, and the optimizer is Adam with weight decay = 0 and beta = (0.9, 0.999). The model’s weights were pretrained on COCO dataset [14].

The mean absolute error (MAE) of MLS was used to evaluate the gap between prediction of the trained model and manually drawing MLS by a clinician, which was defined as below, where *n* is the number of scans, and ${\mathrm{MLS}}_{t}^{\mathrm{GT}}$, ${\mathrm{MLS}}_{t}^{\mathrm{Pred}}$ are ground truth MLS and predicted MLS in mm.

(1) $$\frac{1}{n}\times \overset{n}{\underset{t=1}{\sum }}\left|{\mathrm{MLS}}_{t}^{\mathrm{GT}}-{\mathrm{MLS}}_{t}^{\mathrm{Pred}}\right|$$

## 3. Results

### 3.1. General Characteristics of the Included Patients

A total of 7269 CT slices from 300 patients were included in this study. General characteristics are shown in Table 1. One hundred and sixty-three patients had a single type of hemorrhage (ICH = 60, isolated IVH = 4, SAH = 41, acute SDH = 31, EDH = 1, and chronic SDH = 28), while the other 147 patients had mixed types of hemorrhage. For example, there were 28 patients with only chronic SDH, 16 patients had acute on chronic SDH, and 11 patients with CSDH combing with SAH or ICH in our cohort. Among all CT scanning results, intracranial intraparenchymal hemorrhage was found in 153 CT scans, acute subdural hematoma in 90 scans, chronic SDH in 55 scans, EDH in 5 scans, IVH in 53 scans, and SAH in 115 scans. Ninety-three patients received surgical procedures, and 61 patients received intracranial pressure (ICP) monitoring. The indication for the ICP monitor was acute hemorrhage except for one patient who had acute on chronic SDH with largely acute component. The mean hematoma volume was 42.36 ± 51.03 mL (range 0.002–228.3 mL). For those who received ICP monitoring, the ICP was 18.1 ± 7.9 mmHg on day 0 and 8.9 ± 9.3 mmHg on day 3. The initial GCS was 3–8 in 67 patients, 9–13 in 74 patients, and 14–15 in 159 patients. The Glasgow coma scale on day 3 was 3–8 in 60 patients, 9–13 in 60 patients, and 14–15 in 172 patients. There were 44 deaths on the 30-day outcome, 32 patients with a grade 2 GOS score, 69 patients with a grade 3 GOS score, 141 patients with mild disability, and 10 patients with good recovery. The causes of the hemorrhages are listed in Table 2.

### 3.2. Midline Shift Measurement by Different Methods

Different methods of the MLS measurements are shown in Table 3. There was no significant difference between the different methods, including MLS_MA (measured by the attending neurosurgeon), MLS_R (by the neurosurgical resident doctor), MLS_RA (by a research associate), MLS_GT (calculated from manually selected keypoints), and MLS_Pred (automatic detection method), for the MLS measurements by using ANOVA with Tukey’s comparison (ANOVA *p* = 0.9494, Tukey’s test *p* = 0.9484, >0.999). The intraclass correlation coefficient was 0.9899 (F = 98.589, range, 0.9880–0.9915). The sensitivity and specificity for the detection of MLS greater than 2 mm, 5 mm and 10 mm in the training, validation and testing sets are shown in Table 4. The testing set had a sensitivity of 91.7% and specificity of 80% for the MLS 2 mm cutoff value, 87.5% sensitivity and 96.7% specificity for the MLS 5 mm cutoff value, and 85.7% sensitivity of 97.4% sensitivity for the detection of MLS greater than 10 mm.

### 3.3. Midline Shift and Clinical Outcomes

**Figure 3:**
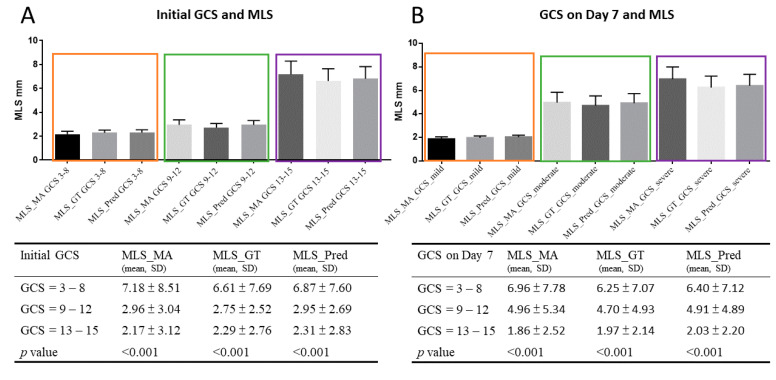
Figure 3 shows the midline shift measured by different methods in different patients with various initial GCSs (**A**) and GCS on day 7 (**B**). The MLS_MA indicates that the MLS was manually measured. MLS_GT represents the MLS measured by the calculation from the automated defined keypoints. MLS_pred is the MLS measurement generated by using the automated detection method. GCS 3–8 (orange), 9–12 (green), and 13–15 (purple) indicates the degree of coma scale in different patients. The MLS was inversely correlated to the coma scale score.

**Figure 4:**
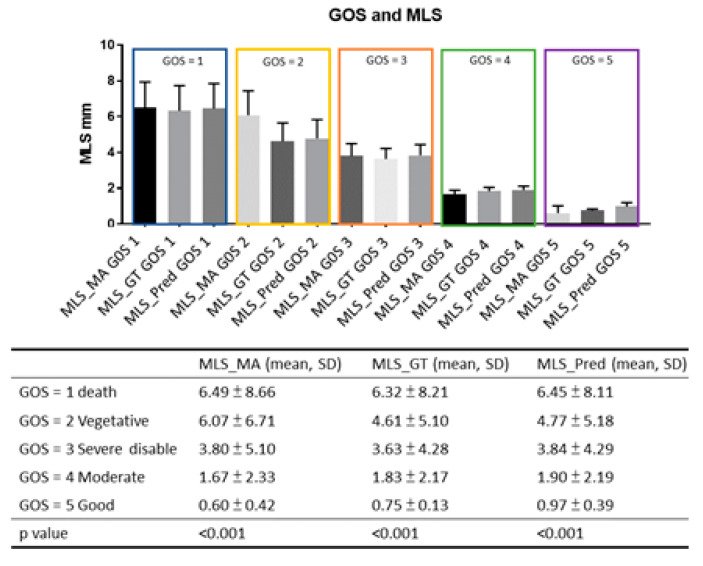
Figure 4 shows the midline shift measured by different methods in different patients with various 30 days Glasgow Outcome Score (GOS). The MLS was inversely correlated to the GOS in all three different measurements (MLS_Yan: manual measurement; MLS_GT: calculated from keypoint detection; MLS_pred: automated MLS detection).

The mean hematoma volume was 42.36 ± 51.03 mL and was highly correlated with the automatic MLS detection results (Pearson r = 0.678, *p* < 0.001). The initial GCS and GCS scores on day 7 and their midline shifts are shown in Figure 3. The degree of the MLS was proportional to the severity of the GCS. The mean MLS was 6.61–7.18 mm in those with initial GCS 3–8, 2.75–2.96 mm in GCS 9–12 and 2.17–2.31 mm in GCS ≥ 13. The mean MLS was 6.25–6.96 in patients with GCS 3–8 at day 7, 4.7–4.96 mm in patients with GCS 9–12 and 1.86–2.03 mm in patients with GCS ≥ 13. The MLS in different GOS scores is shown in Figure 4 and demonstrated an inverse correlation with GOS scores. A summary of the correlation between MLS and clinical outcome is shown in Figure 4. There was no significant correlation between the degree of MLS and the ICP measured in the operation theater and ICP on postoperative day 3.

### 3.4. ROC for Detection of Mortality, Bad Outcomes and Low GCSs

**Figure 5:**
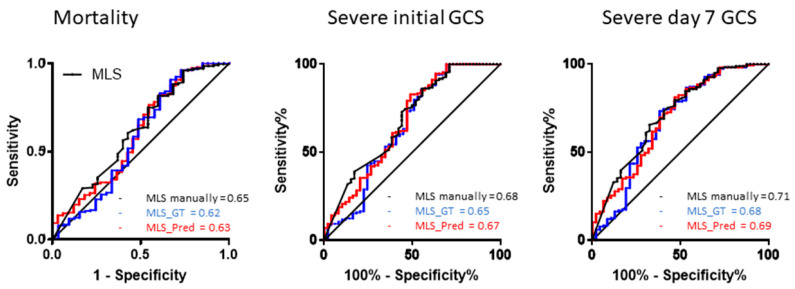
Figure 5 shows the receiver operation curve of MLS measurement by using manual drawing, key point detection and automatic detection methods for the prediction of 30-day mortality and initial and day 7 severe GCS (GCS < 9). There was no difference between different methods.

The prediction of overall 30-day mortality and initial and day 7 severe GCS (GCS < 9) by using the degree of midline shift is shown in Figure 5. The AUCs of MLS using manual drawing, keypoint detection and automatic MLS measurement were 0.68–0.71. There was no difference between different methods.

## 4. Discussion

In this study, we developed a fully automatic detection method for MLS. This method showed high correlation with manual drawing and had fair accuracy in detecting small MLSs (<2 mm) to large MLSs (>10 mm). Detailed clinical prognosis analysis after intracranial hemorrhage was not the main purpose of this study, and our results showed similar clinical correlations in automatic MLS detection and manual drawing.

Manual drawings of the MLS can be biased. In a CT measurement reliability study, the intraclass correlation was approximately 0.652 in one of the study observers [7]. Many automatic or computer-aided MLS detections have recently been developed from symmetry-based, landmarked-based and neural network-based methods [2,15]. In 2010, Liao et al. developed a symmetry-based MLS detection method [8] that showed moderate accuracy in detection accuracy (approximately 80%), and accuracy was low in detecting large MLSs (>5 mm) or spontaneous ICHs. Later, Chen et al. in 2013 used a landmark-based approach and showed that 80% of the detected MLS had less than 2.25 mm error from the actual midline [9]. More recently, Jain et al. in 2019 developed Icobrain, a U-Net-based segmentation and MLS detection method [15]. The authors first used a 2D U-Net-based method for segmentation and second calculated the MLS. In their study of 38 images, the median absolute MLS difference was 0.86 mm with an ICC of 0.93. Our data also showed a mean absolute error of approximately 0.93 mm and ICC = 0.9899 when comparing the automatic detection method and manual drawing. In addition, our data showed good sensitivity (84.6–91.7%) and specificity (80–97.4%) for detecting 2 mm and 5 mm MLSs greater than 10 mm. Therefore, our attempt to use keypoint R-CNN can accurately identify the anterior/posterior falx and the anterior/posterior end of the septum pellucidum rather than to identify the detailed structure of the brain, which can potentially minimize the error and lead to a fair accuracy in MLS detection.

Midline shift has been used as one of the clinical indicators for increased ICP and surgical indications [5,16]. In 2010, Xiao et al. showed a fair accuracy using estimated MLS to predict mortality and good recovery (AUC = 0.752, 0.668). In 2017, Yang et al. investigated 199 spontaneous ICH patients and showed that MLS measured at different levels had a significant correlation with poor clinical outcomes in a multivariable analysis [17]. They also defined that MLS greater than 4 mm can be a predictor for poor outcome. However, another study found an association between MLS and clinical outcome but not a cutoff value for prognosis [4]. A more recent study in 2019 showed that patients with MLS less than 10 mm can have a significant clinical improvement at the 180-day follow-up [18]. Our results showed a similar finding that the degree of MLS was correlated with the initial poor GCS and GCS on day 7 and was inversely proportional to the 30-day GOS score. Our data showed that the AUC for clinical prediction was only approximately 0.7, which was similar to a previous study [19]. Our data did not show a correlation between MLS and ICP on day 0 and day 3, which was not shown in the results. This may be due to several factors. Some patients received ICP monitor placement prior to the decompression, while others received ICP monitor insertion after the decompression. Besides, the measurement of the open pressure after ventricular puncture can be limited and masked by the length of the external ventricular drainage (EVD) tube especially in those with higher ICP. Further the drainage of the EVD in our cohort could be continuous or intermittent which may affect the measurement of the postoperative ICP.

There were several limitations in this study. However, we provided a reasonable size (*n* = 300) containing various types of hemorrhage for the development of the model. The imbalance number in each type of hemorrhage may potentially bias the results. For the clinical applications of MLS, we did not divide the etiology of ICH into subgroups since detailed clinical prognosis analysis was not the main purpose of the study.

## 5. Conclusions

The proposed neural network-based algorithm provides comparable accuracy of MLS detection to that of MLSs manually drawn by clinicians. Such an approach provides a fast, robust, fully automatic method for the detection of MLS, showing its prognostic value for clinical application in the future.

## Figures and Tables

**Table 1:** General Characteristics.

Total Number		300
CT scan (slices)		7269
Age (mean ± SD, range)		48.1 ± 15.1
Types of hemorrhage *	ICH	153
	Acute SDH	90
	Chronic SDH	55
	EDH	5
	IVH	53
	SAH	115
Surgical cases		93
ICP insertion		61
Initial GCS	3–8	67
	9–13	74
	14–15	159
GCS day 7	3–8	60
	9–13	60
	14–15	172
GOS score day 30	1	44
	2	32
	3	69
	4	141
	5	10
Positive pupil reflex, initial	Right (+/−)	244/36
	Left (+/−)	245/41
ICP, day 0 (cmH2O, mean ± SD, range)		18.1 ± 7.9, 0–30
ICP, day 3 (cmH2O, mean ± SD, range)		8.9 ± 9.3, 0–70

* Patients may have mixed types of hemorrhage at the same time.

**Table 2:** Cause of Intracranial Hemorrhage.

Cause of Intracranial Hemoprrhage		
Intraparenchymal Hemorrhage		153
	Trauma	43
	Spontaneous	80
	Secondary *	21
	Postoperative	9
Acute Subdural hematoma (ASDH)		90
	Trauma	68
	Secondary *	18
	Postoperative	4
Subarachnoid Hemorrhage (SAH)		115
	Traumatic	72
	Nontraumatic **	43
Chronic subdural hematoma (CSDH)		55
	Traumatic	43
	Nontraumatic ***	12
Epidural hemorrhage		5
Intraventricular hemorrhage		53

* Secondary causes of hemorrhage included neoplasm, infarction with hemorrhagic transformation, and vascular lesions; ** Nontraumatic SAH included, spontaneous ICH associate SAH, vascular lesion, tumor bleeding, and postoperative changes; *** Nontraumatic CSDH indicated those without clear trauma history.

**Table 3:** Different methods for midline shift measurement.

	MLS MA *	MLS R *	MLS RA *	MLS GT ^#^	MLS Predict ^#^
Slices	300	300	300	6456	7570
Mean (mm)	3.387	3.683	3.639	3.383	3.384
Median	1.300	0.0	2.000	1.490	1.520
Max	29.00	32.00	29.10	25.43	24.49
Min	0.0	0.0	0.0	0.166	0.1987
SD	5.154	5.752	5.224	4.670	4.512
Mean of MAE ^+^				0.213	0.936
Max of MAE				6.227	6.038
CI95 Diff	2.819–3.955	3.050–4.317	3.062–4.216	2.854–3.913	2.872–3.897

No significant difference was observed between different methods for the calculation of the midline shift. * Manually selected the most representative slice from each patient by the attending neurosurgeon (MLS MA), by the resident doctor (MLS R), and by a research associate (MLS RA). ^#^ MLS GT was the results of keypoint calculation, and MLS Predict was calculated by the automatic MLS detection method; ^+^ MAE: mean absolute of error was compared with that of the manual measurement made by the attending surgeon.

**Table 4:** Sensitivity and specificity for detection of midline shift.

	Threshold (mm)	Sensitivity	Specificity
Train	2	89.7%	72.7%
Valid	2	87.5%	85.7%
Test	2	91.7%	80.0%
Train	5	94.0%	96.5%
Valid	5	94.1%	92.9%
Test	5	87.5%	96.7%
Train	10	84.6%	98.5%
Valid	10	83.3%	96.0%
Test	10	85.7%	97.4%

## Data Availability

The data that support the findings of this study are available from the corresponding author, upon reasonable request.
